# Adults’ leisure-time physical activity and the neighborhood built environment: a contextual perspective

**DOI:** 10.1186/s12942-020-00227-z

**Published:** 2020-09-11

**Authors:** Anna Kajosaari, Tiina E. Laatikainen

**Affiliations:** 1 Department of Built Environment, Aalto University, Espoo, PO Box 14100, 00076 Aalto, Finland; 2 Active Life Lab, South-Eastern Finland University of Applied Sciences, Raviradantie 22b, 50100 Mikkeli, Finland

**Keywords:** Physical activity, Public participation GIS, Built environment, Leisure-time physical activity

## Abstract

**Background:**

Context-free outcome measures, such as overall leisure-time physical activity (LTPA), are habitually applied to study the neighborhood built environment correlates of physical activity. This cross sectional study identifies and empirically tests potential methodological limitations related to the use of context-free measures and discusses how these may help in the interpretation of inconsistent associations between participation in moderate-to-vigorous LTPA and objectively measured neighborhood-level built environment attributes.

**Methods:**

We employ a public participation geographic information system (PPGIS), an advanced participatory mapping method, to study the spatial distribution of moderate-to-vigorous LTPA among adult urban Finnish residents (*n* 1322). Secondary sources of GIS land-use and sport facility data were used to disaggregate respondent-mapped LTPA by the behavioral context, such as indoor and outdoor sport facilities, green spaces, and other public open spaces. Associations between the use of the identified LTPA settings and a range of objectively measured neighborhood built environment attributes were studied with multilevel logistic regression models.

**Results:**

Disaggregated by behavioral context, we observed varied and partly opposite built environment correlates for LTPA. The use of indoor and outdoor sport facilities showed no significant associations with their neighborhood availability, but were significantly associated with personal-level attributes. By contrast, participation in LTPA in green and built public open space shared significant associations with access to and availability of neighborhood green space that persisted after controlling for personal-level covariates. Moreover, neighborhood distances up to 1600 m poorly captured participation in moderate-to-vigorous LTPA, as, on average, 40% of visits were located further from home. However, we found the immediate home environment to be an important LTPA setting for the least active participants.

**Conclusions:**

This study demonstrates that LTPA can be a highly heterogeneous measure regarding both the spatial distribution and the environmental correlates of behavioral contexts. The results show that context-free LTPA outcome measures yield inconsistent associations with built environment exposure variables, challenging the applicability of such measures in designing neighborhood-level built environment interventions.

## Introduction

Adults’ physical activity (PA) occurs in several domains within day-to-day life, including active transportation, leisure-time, and occupational and household-based activities [1, 2]. Unlike the other domains of PA, which have relatively clearly defined behavioral contexts, leisure-time physical activity (LTPA) is undertaken in diverse settings and environments [3]. Due to the diversity of potential behavioral contexts, the need to study specific LTPA behaviors in specific settings has been repeatedly voiced in the literature examining correlates and causalities between the physical environment and physical activity outcomes [4–6]. These arguments are founded, on one hand, on the socio-ecological models of health behavior emphasizing the fit between the studied outcome and the environmental levels of influence [2, 7], and, on the other, on empirical results showing stronger correlations between environmental attributes and specific PA behaviors than between these attributes and context-free measures, such as total PA or total LTPA [4].

Despite these recommendations, LTPA outcomes lacking a clearly defined behavioral context are habitually applied in the literature, as demonstrated by a number of recent reviews summarizing the evidence on the built environment correlates of PA [8, 9]. However, while research on the associations between neighborhood built environment characteristics and transport-related PA has established a convincing evidence-base, the relationships between total LTPA and the physical features of the residential environment remain inconclusive [8–12]. In a review of reviews, Choi et al. [8] identified accessibility and population density as the only consistent environmental factors associated with LTPA. A recent meta-analysis, although focused on older adults, reported that total LTPA was most consistently associated with access to sport facilities and access to public open space [3]. Several reviews did not find any consistent associations between overall LTPA and objectively measured environmental features [9, 12]. While associations between total LTPA and the residential environment remain mixed, studies focusing on specific behaviors and behavioral contexts within the leisure-time domain have been able to establish better defined connections with built environment attributes, and, consequently, to produce more reliable evidence to guide and support built environment interventions. Such studies include research investigating the associations between park-based PA and access to and quality of parks and urban green space [13–15], and studies examining correlates between the availability of sport facilities and their use [16].

Travel behavior studies focusing on trip purposes maintain that qualitative destination characteristics influence, in particular, the choice of leisure time destinations, while utilitarian destinations, such as grocery stores or other daily services, are more likely to be chosen based on distance to the main nodes of everyday life [17, 18]. Yet, studies examining associations between the availability of recreational opportunities and an outcome measure of LTPA generally operate under the assumption that proximity is a key determinant of LTPA destination choice. Distance to the nearest facility from home and the density of facilities within a pre-defined buffer distance are frequently applied to operationalize proximity to PA facilities [19]. However, these measures may overly generalize the leisure-time destination choice by prioritizing proximity over other, symbolic and qualitative, destination qualities that may impact destination choice [17, 20].

Regarding LTPA destinations, the main qualitative differences concern the activities facilitated by these settings, allowing differentiation between, for instance, recreational facilities for specific sports, or urban open space supporting recreational walking. As a qualitative attribute, the specialization of the activity is directly related to the size of the destination’s catchment area, i.e., the geographical area attracting its potential users. Consequently, a specialized LTPA destination is likely to attract users willing to increase their travel distance and time where such facilities are unavailable closer to home. In urban contexts, recreational facilities can be identified based on whether their provision extends to neighborhood, local, or city-wide level [21, 22]. In addition to destination type, qualitative aspects of LTPA destinations may, for instance, include positive environmental or cultural perceptions and social interactions. The latter, for its part, may require the negotiation of destination choice between individuals living in different areas, thus favoring central locations with high regional accessibility.

Existing evidence on adults’ leisure-time travel behavior supports the above notions on LTPA destination choice. Studies on adults’ travel distances to diverse LTPA destinations consistently find that travel distances vary both in relation to personal and destination characteristics [23]. Moreover, studies focusing on contextual exposures to the built environment propose that LTPA is not necessarily undertaken in the residential environment, and is thus less influenced by built environment features measured on this scale [12, 24–26]. This assumption is supported by consistent empirical evidence from studies applying GPS and accelometer methods to spatially locate PA, reporting high level of adults’ overall PA undertaken outside of threshold neighborhood distances varying from 800 to 1600 m [24, 27–29]. However, considering the diversity of LTPA behaviors, it is likely that the neighborhood environment is more important for some activities than for others. Prior research findings suggest that within the LTPA domain, recreational walking, in particular, is correlated with the neighborhood environment [30].

Moreover, defining the context of individuals’ PA behavior based with administrative or buffer-based boundaries, such as postal code areas, census tracts, and home buffers has been proven problematic [31]. These approaches tend to assume that individuals are exposed solely to the environment around their residency and, consequently, do not capture the context outside the applied neighborhood boundaries. The modifiable areal unit problem (MAUP) and the uncertain geographic context problem (UGCoP) pose key challenges to researchers examining the associations between the physical environment and PA [31]. MAUP is linked to the problems in capturing the areal unit of analysis at differing spatial scales or with varying criteria whereas UGCoP depicts the uncertainty of the researcher-defined unit of analysis in capturing the spatio-temporal realities of actual human health behavior [32, 33]. However, there are multiple examples of recent research applying more versatile and multidimensional analyses to capture individuals’ actions within and outside their neighborhoods to overcome these challenges [34–36].

### Study objective

We can derive two main conclusions from the previous discussion on problems occurring when studying the associations between neighborhood-level built environment attributes and a context-free LTPA measure. First, these measures are bound to comprise of heterogeneous behavioral contexts. This is likely, on one hand, to hide processes impacting participation in LTPA, and on the other, to reduce the validity of the observed associations. Second, a context-free outcome variable, such as overall LTPA, may include behavioral contexts chosen based not only on their location but also on unmeasured qualitative or symbolic characteristics. Consequently, built environment attributes measured on the level of the residential environment do not necessarily capture visits to specialized sports facilities with larger catchment areas. Therefore, uncertainty in the behavioral context challenges the meaningfulness of the information residential environment correlates convey about participation in LTPA.

This paper draws attention to the challenges of studying LTPA outcomes without specifying the behavioral context. The following sections proceed to empirically test the above mentioned limitations by analyzing adult urban dwellers’ moderate-to-vigorous LTPA behavior with primary spatial data on the distribution of these activities. We examined the type, spatial distribution, and visitation patterns of diverse settings for moderate-to-vigorous LTPA, and analyzed which built environment and individual-level characteristics affected LTPA destination choice. Public Participation GIS (PPGIS) method [37] was employed to facilitate large-scale data collection including both spatial data on LTPA and a range of intrapersonal attributes related to the study participants. This approach allowed us to follow a socio-ecological framework [2, 7] addressing the multiple levels of influence on physical activity behavior. Finally, the last sections summarize key findings and discuss implications for policy and practice.

## Methods

### Data collection

The data were collected between August and September 2018 in the Helsinki Metropolitan Area, Finland. This area consists of the municipalities of Helsinki, Espoo, Vantaa, and Kauniainen and is the largest urban area in Finland with a population of 1,2 million inhabitants [38]. A simple random sample of 10,000 working-age (18–65 years) adults living permanently in the study area was ordered from the Finnish Population Register Centre. These sample members received a letter of invitation to participate in the online survey, followed after two weeks by a reminder post card.

**Fig. 1:**
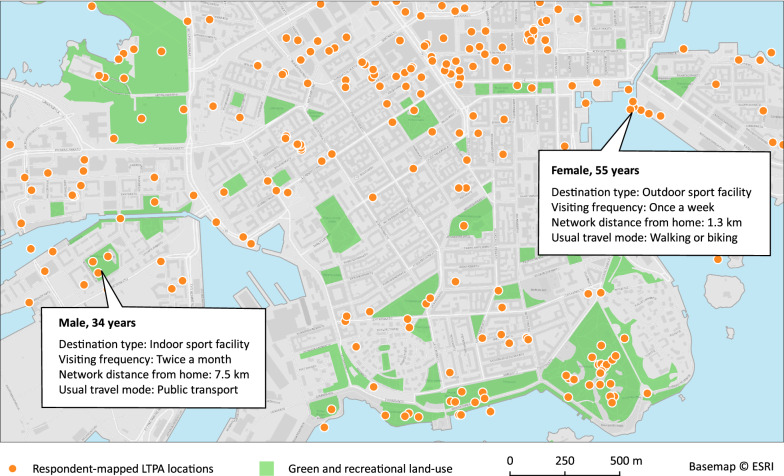
Distribution of respondent-mapped places for moderate-to-vigorous LTPA in South Helsinki. Call-out boxes exemplify the features’ attribute data

The respondents were instructed to think of all the places that they usually visit for moderate-to-vigorous LTPA in the time of the year of the data collection, and to locate them in the survey’s mapping view. The respondents answered to follow-up questions concerning each mapped location, reporting the approximate visiting frequency, usual activity level (moderate or vigorous PA), and whether the activity took place indoors or outdoors (Fig. 1). The respondents had the possibility to map their activity either as a point or a polyline feature. In addition to the mapping tasks, the survey included sections on socio-economic and demographic background, self-efficacy and social support for PA, and PA behavior.

Altogether 1583 respondents participated in the survey resulting in a response rate of 16%. The final sample of this study consisted of those 1322 respondents who had mapped their residential location and had complete information on LTPA items. The demographic and socio-economic characteristics of the survey respondents were compared to data from the Helsinki Metropolitan Area [38, 39]. The data showed general consistency on most socio-economic and demographic variables within the study area. The participants were 57% female compared to 51% of the same age group in the study area. Participants with higher levels of formal education were over-represented, comprising 51% of the participants compared to 37% in the Helsinki Metropolitan Area. Also, age groups from 50 to 65 years were slightly over-represented.

The respondents mapped altogether 3507 individual locations for moderate-to-vigorous LTPA. Locations situated outside of the Greater Helsinki Region were removed from the sample, resulting in the sample of 3408 features, from which 63% were mapped as points and 37% as routes. The starting points of the route features were extracted for the analytical purpose of this study. Distances from the residential location to points and route starting points were measured as shortest path street-network distance. Distances to features located in green areas were calculated to the closest street segment. A continuous estimate of the monthly visits to each destination was calculated as follows: “every day”, 30; “nearly every day”, 20; “a couple of times a week”, 8; “once a week”, 4; “a couple of times a month”, 2; and “once a month”, 1.

### Measures

#### Settings for moderate-to-vigorous LTPA

The environment type of each mapped activity location was determined using overlay analyses with secondary sources of GIS land-use and sport facility data. In order to increase specificity measuring public open space [40], we distinguished between activities undertaken in green public open spaces and in non-green public open spaces, such as streets, walkways, and other built-up and publicly maintained areas. Sport facilities in indoor and outdoor settings were identified separately. Consequently, the following four LTPA settings were identified:

- *Indoor sports facilities,* including points mapped as indoor locations and situated within 150 m of an indoor sport facility. These were identified using LIPAS, a national database of sport facilities developed and administered by the University of Jyväskylä. The remaining indoor points (*n* 125, 17% of all indoor locations) were identified as LTPA undertaken in private indoor settings (e.g., home, workplace) and were removed from the analysis.
- *Outdoor sport facilities*, including points located in sport and recreational land-use, such as sport fields, outdoor gyms, etc. (Topographic database 2018, National Land Survey of Finland), and points located within 50 m from individual outdoor sport facilities (University of Jyväskylä, LIPAS sport facility database).
- *Green public open space*, including points located within and routes predominantly located in forest and semi-natural areas, farmlands, and urban parks (CORINE Land Cover 2018, classes 141 (level 3), 2, and 3 (level 1)).
- *Built public open space,* including points located within and routes predominantly located in residential areas, walkways, and other non-green public open space.

Two dichotomic outcome variables were created for each of the identified environmental settings based on whether the respondent had mapped a place for moderate-to-vigorous LTPA visited (1) at least once a week or more often, or (2) at least once a month or more often.

#### Built environment characteristics

The built environment features of the residential area were measured within an 800 m and 1600 m Euclidean buffer distance from the residential location. Eight hundred meters corresponds to roughly a 10–15 min walking distance, and is among the most commonly applied neighborhood distances in studies assessing contextual exposure to the residential environment in physical activity studies [41]. Due to the good availability of sport facilities in the study area, a lower cut-off value of 400 m was used to measure distance to the closest sport facility. The following built environment variables were included in the analyses:

- *Density of sport facilities* The number of indoor sport facilities (e.g., private and public gyms, indoor swimming pools, sports halls, etc.) and outdoor sport facilities (e.g., neighborhood sports facilities, sports fields and courts, etc.) within the neighborhood buffer were calculated separately (University of Jyväskylä, LIPAS sport facility database).
- *Proximity to sport facilities* Dichotomic variables were formed to indicate, whether the respondent had access to at least one indoor or outdoor sport facility within 400 m from home.
- *Availability of public green space* The area of public green space within the neighborhood buffer was calculated from CORINE Land Cover dataset, including parks, natural environments, and agricultural landscape (CORINE Land Cover 2018, L3 141, 211–421).
- *Proximity to public green space* Additionally, dichotomic variables were formed measuring access to at least one middle-sized (> 30 ha) and one large (> 100 ha) green area within the threshold distances of 400 m, 800 m, and 1600 m.
- *Residential density,* measured as the ratio of the residential floor area to the land area in residential use.

Summary statistics of the built environment variables are provided in Table 1.

**Table 1:** Built environment and psychosocial measures

	Total sample (*n* 1322)
Built environment characteristics	
Number of indoor sport facilities, mean (SD)	
800 m	10.3 (9.9)
1600 m	33.3 (27.7)
Distance to the closest indoor sport facility (%)	
≤ 400 m	68.5
Number of outdoor sport facilities, mean (SD)	
800 m	10.6 (6.7)
1600 m	36.4 (17.1)
Distance to the closest outdoor sport facility (%)	
≤ 400 m	78.6
Green area km^2^, mean (SD)	
800 m	0.6 (0.3)
1600 m	2.4 (1.1)
Access to green area over 100 ha (%)	
≤ 400 m	27.0
≤ 800 m	45.0
≤ 1600 m	71.6
Access to green area over 30 ha (%)	
≤ 400 m	51.1
≤ 800 m	76.9
≤ 1600 m	93.2
Residential density, mean (SD)	
800 m	0.6 (0.5)
1600 m	0.5 (0.4)
Psychosocial measures	
Self-efficacy, mean (SD)	3.2 (0.8)
Social support, mean (SD)	2.8 (0.9)

*SD* standard deviation

#### Intrapersonal variables

Moderate-to-vigorous LTPA, expressed in Metabolic Equivalent Task (MET) minutes, was measured with the LTPA module of the International Physical Activity Questionnaire (IPAQ) [42], long form. Respondents were divided into quartiles based on the moderate-to-vigorous LTPA MET-minutes accumulated during a usual week. The impact of psychosocial factors on LTPA behavior was studied with self-efficacy and social support for PA. Both variables have been associated in previous studies with an increase in PA [8, 10, 43–45]. Social support for PA was measured with three items based on the Social Support for Exercise Scale [46] (Additional file 1). The scale was calculated as the mean score of three items, and showed good internal consistency (α = 0.78). Self-efficacy for PA was measured as the respondent’s confidence in being physically active when met with internal or external challenges, such as being tired or bad weather. Respondents indicated agreement with five statements modified from Sallis et al. [47] on a 5-point Likert scale. The scale had good internal consistency (α = 0.73).

### Statistical analyses

The differences between LTPA settings were assessed with Chi-square and Kruskal–Wallis H tests. Multilevel logistic regression models were used to study associations between participation in moderate-to-vigorous LTPA in different environmental settings and the built environment characteristics. Multilevel analyses were used to account for clustering of the data at the postal code area level. All analyses were performed with IBM SPSS Statistics v26 and were adjusted for gender, age, education level, employment status, total amount of self-reported moderate-to-vigorous LTPA, social support for PA, and self-efficacy for PA. Independent variables with skewed distributions (moderate-to-vigorous LTPA, residential density, availability of public green space) were log transformed for analysis.

## Results

### Descriptives of LTPA settings and visitation patterns

Public green spaces (41% of mapped locations) and built public open spaces (32% of locations) were the most common settings for moderate-to-vigorous LTPA, followed by indoor (19%) and outdoor sport facilities (8%). A considerable share of the monthly visits to these locations took place outside of the applied 800 m (63%) and 1600 m (40%) threshold distances (Table 2). However, the average network distances from home to places for LTPA (*H*(3) = 423.1, *p* < 0.001) as well as the visiting frequency (*H*(3) = 22.9, *p* < 0.001) both varied significantly between the LTPA settings. LTPA locations situated in green and built public open spaces were on average accessed closest to home, with 56% and 69% of the mapped locations, respectively, situated within the neighborhood distance of 1600 m. On the other hand, indoor and outdoor sport facilities were on average accessed further from home, with only 27% of indoor sports facilities located within 1600 m. Moreover, visiting frequency correlated negatively with network distance from home (r = -0.191, *p* < 0.001), indicating that locations situated closer to home were visited more frequently.

**Table 2:** Descriptives of respondent-mapped locations for moderate-to-vigorous LTPA

	*n* (%)	LTPA locations						Monthly visits to LTPA locations		
		Locations/respondent, mean	≤ 800 m from home (%)	≤ 1600 m from home (%)	Distance from home (km), mean	Moderate PA (%)	Vigorous PA (%)	Visits/respondent, mean	≤ 800 m from home (%)	≤ 1600 m from home (%)
Total	3305 (100.0)	2.5	33.1	52.6	3.2	62.5	37.5	7.8	36.8	60.0
Indoor sport facility	620 (18.8)	0.5	10.5	27.3	4.7	27.4	72.6	6.5	11.4	28.9
Outdoor sport facility	271 (8.2)	0.2	13.7	38.7	3.9	58.6	41.4	6.9	16.8	48.7
Public green space	1345 (40.7)	1.1	33.2	55.9	3.2	72.4	27.6	7.7	38.1	66.4
Built public open space	1069 (32.3)	0.9	52.1	69.1	2.1	77.3	22.7	9.3	56.4	76.0
*p-*value		< 0.001	< 0.001	< 0.001	< 0.001	< 0.001	< 0.001	< 0.001	< 0.001	< 0.001

Chi–square tests were used for categorical variables and Kruskal–Wallis H tests for continuous variables

The usual activity level varied significantly between the LTPA settings (χ^2^ = 441.6, *p* < 0.001). The proportion of places where physical activities were performed at a vigorous level was highest among indoor and outdoor sport facilities, 73% and 41%, respectively. Green and built public open spaces were predominantly used for moderate PA, which was identified as the usual activity level for 72% and 77% of the mapped locations, respectively.

Significant in-group differences existed between sample subgroups stratified by personal characteristics and the use and distribution of places visited for moderate-to-vigorous LTPA (Table 3). The level of self-reported moderate-to-vigorous LTPA showed the most consistent differences, as groups with the highest amount of LTPA had a significantly higher monthly visiting frequency in LTPA destinations (*H*(3) = 97.3, *p* < 0.001). Furthermore, both the proportion of individual LTPA locations and the number of visits to LTPA locations outside the applied neighborhood distances were significantly higher for respondents with the highest self-reported LTPA. On average, 44% of visits by respondents in the lowest LTPA quartile were within 800 m of their home and 69% within 1600 m. By contrast, the figures for respondents in the highest quartile were 32% within 800 m of their home and 53% within 1600 m.

**Table 3:** Sample characteristics and spatial distribution of moderate-to-vigorous LTPA

	*n* (%)	LTPA locations			Monthly visits to LTPA locations		
		Locations/respondent, mean	≤ 800 m from home (%)	≤ 1600 m from home (%)	Visits/respondent, mean	≤ 800 m from home (%)	≤ 1600 m from home (%)
Total	1322 (100.0)	2.4	33.1	52.6	18.7	36.8	60.0
Gender							
Female	758 (57.3)	2.4	34.4	55.2	19.0	37.9	60.9
Male	550 (41.6)	2.3	31.2	49.0	18.3	34.9	58.4
* p-*value		0.143	0.164	0.047	0.123	0.906	0.333
Age (years)							
18–29	319 (24.1)	2.5	36.3	55.8	18.5	36.7	59.5
30–39	276 (20.9)	2.4	39.3	57.2	18.4	45.6	65.4
40–49	239 (18.1)	2.4	30.0	49.0	17.0	31.7	55.6
50–59	310 (23.4)	2.4	30.1	51.8	19.7	34.8	61.4
60–66	172 (13.0)	2.3	25.9	45.7	19.7	31.5	53.8
* p-*value		0.130	< 0.001	0.028	0.301	< 0.001	0.004
Employment status							
Employed	781 (59.1)	2.4	33.2	52.4	18.9	37.5	60.3
Student	172 (13.0)	2.7	37.0	54.8	17.7	39.0	58.7
Other	165 (12.5)	2.6	31.5	51.5	23.2	35.1	61.7
* p-*value		0.179	0.226	0.390	0.007	0.051	0.190
Educational level							
University degree	674 (51.0)	2.7	34.9	53.1	18.8	38.5	60.7
Lower	437 (33.1)	2.2	31.7	52.9	20.1	35.7	59.9
* p-*value		< 0.001	0.149	0.705	0.823	0.007	0.040
Moderate-to-vigorous LTPA (MET-minutes/week)							
Q1 (< 477 MET-minutes)	331 (25.0)	1.6	38.2	61.2	14.4	44.4	68.8
Q2 (477–1200 MET-minutes)	330 (25.0)	2.5	31.3	51.8	16.5	34.3	62.3
Q3 (1201–2100 MET-minutes)	330 (25.0)	2.6	35.1	53.8	18.1	40.4	61.6
Q4 (> 2100 MET-minutes)	331 (25.0)	3.1	30.1	47.6	25.6	32.0	52.9
* p-*value		< 0.001	0.047	< 0.001	< 0.001	0.007	< 0.001

Chi–square tests were used for categorical variables and Kruskal–Wallis H tests for continuous variables

Because of missing data, all percentages do not equal 100%

Neither the number of mapped locations nor the number of visits varied significantly between respondent groups stratified by gender or age. Respondents with a university-level degree mapped on average more individual places for LTPA than those with lower degrees. In addition, a significant difference existed between age and distribution of LTPA, as, on average, respondents in the younger age groups mapped places for LTPA closer to home.

### Associations between built environment attributes and use of LTPA settings

The adjusted odds of engaging in moderate-to-vigorous LTPA in different environmental settings at least once a week and at least once a month are presented in Table 4. The associations between participation in moderate-to-vigorous LTPA in diverse settings and the studied environmental characteristics varied greatly for both outcome measures. In general, LTPA undertaken in both green and built public open spaces was more consistently associated with built environment characteristics than was LTPA in specialized indoor or outdoor sport facilities, which was more strongly associated with intrapersonal attributes.

**Table 4:** Adjusted odds of participating in moderate-to-vigorous LTPA in indoor or outdoor sport facilities, public green space, and other public open space

	Visited at least once a week								Visited at least once a month							
	Indoor sport facility		Outdoor sport facility		Public green space		Other public open space		Indoor sport facility		Outdoor sport facility		Public green space		Other public open space	
	OR	(95% CI)	OR	(95% CI)	OR	(95% CI)	OR	(95% CI)	OR	(95% CI)	OR	(95% CI)	OR	(95% CI)	OR	(95% CI)
Number of indoor sport facilities																
800 m	1.00	(0.99–1.02)	1.00	(0.99–1.03)	0.98*	(0.97–0.99)	1.01	(0.99–1.02)	1.01	(0.99–1.02)	1.01	(0.99–1.03)	0.98**	(0.96–0.99)	1.01	(0.99–1.02)
1600 m	1.00	(0.99–1.01)	1.00	(0.99–1.02)	0.99*	(0.98–0.99)	1.01	(0.99–1.01)	1.01	(0.99–1.01)	1.00	(0.99–1.01)	0.99**	(0.98–0.99)	1.01*	(1.00–1.01)
Distance to the closest indoor sport facility																
≤ 400 m (ref. > 400 m)	0.99	(0.73–1.35)	0.99	(0.75–1.74)	1.02	(0.76–1.37)	1.27	(0.95–1.71)	0.88	(0.65–1.18)	1.05	(0.72–1.52)	0.94	(0.70–1.26)	1.29	(0.98–1.70)
Number of outdoor sport facilities																
800 m	1.01	(0.99–1.04)	1.01	(0.99–1.05)	1.00	(0.98–1.02)	1.02	(0.99–1.04)	1.02	(0.99–1.04)	1.03	(0.99–1.05)	0.99	(0.97–1.02)	1.02	(0.99–1.04)
1600 m	1.01	(0.99–1.01)	1.01	(0.99–1.02)	1.00	(0.98–1.01)	1.01	(0.99–1.01)	1.01	(1.00–1.02)	1.01	(0.99–1.02)	1.00	(0.99–1.01)	1.01	(0.99–1.01)
Distance to the closest outdoor sport facility																
≤ 400 m (ref. > 400 m)	0.94	(0.67–1.31)	0.94	(0.80–2.11)	0.94	(0.68–1.30)	1.13	(0.81–1.57)	0.95	(0.68–1.32)	1.14	(0.75–1.74)	1.05	(0.76–1.45)	1.26	(0.92–1.71)
Green area m^2^																
800 m	0.82	(0.48–1.39)	0.82	(0.31–1.24)	4.42***	(2.50–7.80)	0.34***	(0.21–0.56)	0.91	(0.54–1.54)	0.76	(0.40–1.45)	5.41***	(3.15–9.29)	0.39***	(0.25–0.63)
1600 m	0.82	(0.44–1.55)	0.82	(0.21–1.04)	4.40***	(2.50–8.59)	0.26***	(0.15–0.46)	0.82	(0.44–1.54)	0.56	(0.26–1.19)	5.03***	(2.67–9.47)	0.26***	(0.15–0.45)
Access to green area over 30 ha																
≤ 400 m (ref. > 400 m)	1.03	(0.77–1.36)	1.03	(0.73–1.58)	1.81***	(1.36–2.40)	0.56***	(0.43–0.73)	1.15	(0.87–1.53)	1.24	(0.87–1.76)	1.93***	(1.46–2.55)	0.64**	(0.50–0.82)
≤ 800 m (ref. > 800 m)	1.29	(0.90–1.84)	1.29	(0.55–1.37)	1.54*	(1.07–2.22)	0.53***	(0.38–0.72)	1.33	(0.93–1.89)	1.05	(0.68–1.63)	1.69**	(1.19–2.38)	0.62**	(0.46–0.84)
≤ 1600 m (ref. > 1600 m)	0.88	(0.49–1.58)	0.88	(0.51–2.82)	0.99	(0.54–1.82)	0.50**	(0.30–0.85)	0.90	(0.50–1.61)	1.14	(0.53–2.45)	1.16	(0.64–2.09)	0.55*	(0.32–0.93)
Access to green area over 100 ha																
≤ 400 m (ref. > 400 m)	0.97	(0.71–1.33)	0.97	(0.58–1.38)	1.98***	(1.45–2.70)	0.51***	(0.37–0.71)	1.01	(0.74–1.38)	1.01	(0.68–1.48)	2.01***	(1.46–2.75)	0.59***	(0.44–0.78)
≤ 800 m (ref. > 800 m)	0.97	(0.73–1.30)	0.97	(0.51–1.16)	1.79***	(1.34–2.40)	0.59***	(0.45–0.78)	1.00	(0.75–1.33)	0.79	(0.55–1.12)	1.70***	(1.28–2.25)	0.58***	(0.45–0.75)
≤ 1600 m (ref. > 1600 m)	1.12	(0.80–1.56)	1.12	(0.45–1.03)	1.56**	(1.11–2.19)	0.69*	(0.51–0.93)	1.15	(0.83–1.60)	0.76	(0.51–1.13)	1.75***	(1.28–2.40)	0.65**	(0.49–0.87)
Residential density																
800 m	1.15	(0.76–1.74)	1.15	(0.98–2.93)	0.81	(0.52–1.25)	1.34	(0.90–1.99)	1.25	(0.83–1.88)	1.56	(0.94–2.61)	0.76	(0.49–1.16)	1.59*	(1.11–2.30)
1600 m	1.20	(0.74–1.93)	1.20	(0.99–3.40)	0.66	(0.40–1.10)	1.58*	(1.01–2.47)	1.42	(0.89–2.26)	1.66	(0.93–2.96)	0.63	(0.38–1.03)	1.68*	(1.11–2.54)

All models accounted for clustering at the postal code area level and controlled for age, gender, education (university degree vs. lower), employment (employed vs. other), self-efficacy, social support, and physical activity level

*p  < 0.05, **p  < 0.01, ***p  < 0.001, *OR* odds ratio, *CI* confidence interval

No significant associations were observed between the exposure variables and LTPA in outdoor or indoor sport facilities. However, an increase in moderate-to-vigorous LTPA raised the likelihood of participating in LTPA in both indoor and outdoor sport facilities after adjusting for the other intrapersonal and environmental characteristics (see Additional file 2 for full models including age, gender, education, employment, self-efficacy, social support, and physical activity level). Males had higher odds of participating in weekly or monthly moderate-to-vigorous LTPA in outdoor sport facilities than females. Females, full-time employed respondents, and respondents with a university-level degree had higher odds of participating in LTPA in indoor sports facilities.

Participation in moderate-to-vigorous LTPA in public green spaces was associated with availability and proximity to public green space. The amount of green space was positively associated with an increased likelihood of weekly LTPA in green environments measured both in 800 m (OR: 4.42, *p* < 0.001) and in 1600 m buffers (OR: 4.40, *p* < 0.001), and the likelihood of monthly LTPA in 800 m (OR: 5.41, *p* < 0.001) and in 1600 m buffers (OR: 5.03, *p* < 0.001). Living close to a large green area (> 100 ha) significantly increased the odds of LTPA in green spaces in all threshold distances, while proximity to a middle-sized (> 30 ha) green area increased the odds only when accessible within 400 m or 800 m from home. Negative associations existed between participation in LTPA in green spaces and the density of indoor sport facilities in the neighborhood. These results are likely explained by significant (*p* < 0.001) negative correlations between amount of green space and this variable in both 800 m and 1600 m buffer distances. In addition, an increase in moderate-to-vigorous LTPA increased the odds of weekly, but not monthly, use of green LTPA environments. An increase in social support for PA and having a university-level degree both increased the odds of participating in LTPA in green spaces at least once a month. The likelihood of participating in LTPA in built public open spaces significantly decreased with the availability and access to green space in the neighborhood. The odds of using built public open spaces for LTPA at least once a month increased with residential density and the availability of indoor sport facilities, which correlated with residential density. Being female and an increase in age also raised the likelihood of exercising in these environments.

We did not observe any significant associations between participation in moderate-to-vigorous LTPA (measured in MET-minutes as the outcome variable, multilevel linear model) and the density of indoor or outdoor sports facilities, access to green space in 400 m, 800 m or 1600 m, or residential density or availability of green space within 800 m and 600 m neighborhood distances (analyses not included).

## Discussion

Healthy cities promote easy and equitable access to places where residents of diverse socio-economic background, life stages, and physical ability may engage in health-supportive and health-enhancing LTPA. With increasing research interest towards correlates between adults’ PA and the neighborhood built environment [48], this study aimed to highlight the limitations of studying LTPA outcomes without specifying the behavioral context. By disaggregating LTPA by behavioral context, we have demonstrated that LTPA can be a highly heterogeneous measure regarding both the spatial distribution and the environmental correlates of behavioral contexts. These results help to contextualize prior studies reporting mixed or weak relationships [8–10, 12] between participation in LTPA and the neighborhood built environment.

### LTPA settings

Our study assessed the impact of built environment attributes on the likelihood of participating in moderate-to-vigorous LTPA in diverse environmental settings independently from intrapersonal attributes, such as socioeconomic background, individual activity level, and social support and self-efficacy for PA. Separated by behavioral context, we observed varied and partly opposite built environment correlates for LTPA. The use of indoor and outdoor sport facilities did not show significant associations with their neighborhood availability, but were significantly associated with intrapersonal attributes, including moderate-to-vigorous LTPA and social support for PA. In addition, full-time employed participants and participants with a university level degree were more likely to engage in weekly or monthly LTPA in indoor sports facilities. This result is in line with prior studies reporting increased odds of using sport facilities for higher income and education groups [16] and cost as a key barrier for using sport facilities [49].

Overall, the results on the effect of the neighborhood availability of sport facilities and their use suggest that besides proximity, other variables direct destination choice for LTPA in sport facilities. As discussed earlier in this paper, these are likely to be related to destination type and quality. By contrast, participation in moderate-to-vigorous LTPA in public open spaces shared strong associations with environmental attributes that persisted after controlling for intrapersonal variables. In particular, participation in LTPA in green and natural environments increased with the neighborhood availability of green space and access to middle-sized and large green areas within walking distance. However, the neighborhood availability of green space decreased the likelihood of LTPA in other public open spaces. These results are in line with prior studies reporting access to parks and urban green spaces to be associated with park-based PA [13–15], but not necessarily with overall PA [14].

Moreover, some built environment attributes provided significant, yet opposite associations depending on the LTPA setting. Such results highlight the challenges of interpreting environmental correlates of global outcome measures, as it is likely that these opposite associations are not manifested when an outcome measure combining different behavioral contexts is used. Alternatively, associations between overall LTPA and the neighborhood built environment may be detected, but present only for those respondents whose LTPA consists mostly of neighborhood-based activities. In both situations, a context-free outcome variable offers limited understanding of the ways in which residential environments support LTPA.

Furthermore, we observed some unexpected associations between built environment attributes and the use of certain LTPA settings. For example, the density of indoor sport facilities increased the likelihood of LTPA in built public open spaces. This result suggests that, in unadjusted models, such variables as the availability of sport facilities might serve as a proxy for a correlated attribute associated with recreational walking, such as land-use mix or residential density, indicating high destination availability. The reliability of such models can be increased by establishing clear hypotheses on the interaction between the outcome behavior and the built environment attribute. However, models lacking knowledge of the behavioral context, as is the case with those applying global outcome measures such as overall LTPA or overall PA, remain unable to test and verify relationships between built environment attributes and specific LTPA behaviors.

### Spatial distribution of LTPA

Neighborhood distances of 800 m and 1600 m poorly captured population-level participation in moderate-to-vigorous LTPA, as, on average, 63% and 40% of visits to LTPA destinations, respectively, were located further from home. This result is consistent with prior GPS-studies locating adults’ PA in urban settings [24, 27–29]. However, the average distances from home varied considerably between different LTPA settings. Public green spaces, including public parks and natural environments, and other public open spaces, such as walkways and non-green urban space, were on average accessed closer to home than specialized sport facilities. In addition, the majority of activities in these settings were undertaken on a moderate activity level. These results are in line with prior research finding neighborhood-centric built environment measures to be associated with recreational walking [50–52] and park-based PA [14, 15]. Indoor sport facilities were, on average, the least often accessed close to home, confirming that typical neighborhood threshold distances do not necessarily capture visits to specialized sports facilities with larger catchment areas. Considering the wider distribution of these destinations, population-level studies on the accessibility of sport facilities might benefit from a larger scale of analysis [53, 54] or an extended focus on the other environments of the daily life, such as the work place and other frequently visited destinations [25]. The latter approach has been increasingly applied in studies questioning the suitability of neighborhood-based exposure measures to study PA [26, 55] or other health behavior outcomes, such as associations between food environments and food purchasing behaviors [56, 57].

However, the share of neighborhood moderate-to-vigorous LTPA varied considerably between population sub-groups. Participants with the lowest levels of moderate-to-vigorous LTPA tended to concentrate their activities in the vicinity of the residential location and on a moderate activity level, and were active particularly in green and built public open spaces. By contrast, the most active individuals were more likely to engage in LTPA outside the neighborhood. These results suggest that the residential environment provides an important setting for moderate, health-supportive LTPA, and that neighborhood environments supporting moderate-level LTPA, such as walking for leisure, can be particularly beneficial for sustaining the activity levels of groups with low LTPA. Previous studies have also shown the central role of the supportiveness of the neighborhood environment for PA [58], especially among groups who are the most affected by the characteristics of their neighborhoods, such as older adults [59].

Overall, our results suggest that the provision of LTPA facilitating characteristics, such as mid- or large sized green areas, can create more opportunities to be active in the immediate home vicinity, especially for the least active population segments. However, it should be noted that the mere provision of these characteristics alone might not yield the expected results; in addition, the removal of potential physical environmental barriers, such as heavy traffic, poorly maintained infrastructure, or inadequate lighting, should be also considered. Removal of environmental barriers to enhance LTPA might function as an additional solution to the provision of LTPA facilitating characteristics especially in the most vulnerable communities and neighborhoods [60].

### Implications for practice, policy, and future research

Despite recommendations to increase the fit between environmental attributes and the studied health behavior [4–6], overall measures of LTPA with unspecified behavioral contexts are habitually applied in the literature. Possible reasons for this include reliance on survey data differentiating between the domains of PA without specifying the behavioral context, as well as the increased availability of secondary GIS data enabling the analysis of objectively measured built environment features.

Turning to recreational and transport geography, we have argued that behaviors within the LTPA domain differ not only by the type of activity, but also by their environmental correlates, spatial distribution, and the process of destination choice. Disaggregating self-reported moderate-to-vigorous LTPA by context, our results show that context-free LTPA measures can include, on one hand, activities that are mostly related to intrapersonal variables, and on the other, those that are strongly related to LTPA opportunities in the neighborhood environment. Studies focusing solely on overall LTPA are likely to under- or overestimate these processes depending on the composition of overall LTPA. Likewise, variation in the behavioral contexts comprising LTPA may partly explain why the observed environmental correlates of overall LTPA vary between studies. These problems can be mitigated by matching the studied environment and behavior, for example by studying the neighborhood correlates of within-neighborhood LTPA [3, 4]. In addition, we recommend establishing clear arguments for why certain spatial analysis levels are expected to influence the studied behavior.

Whereas the literature on the built environment associations of physical activity recommends separating different domains such as LTPA and active travel analytically and theoretically [1, 2], the results of this study show that, on a population level, LTPA is likely to consist of diverse environmental and behavioral contexts, and linking it to neighborhood built environment characteristics is challenging. Studies focusing on environmental correlates of LTPA would benefit from using analyses that separate between distinct behavioral settings, such as green public open spaces, built-up public open spaces, and diverse sport facilities. While total measures of LTPA are necessary to compare and study population and individual level changes in LTPA, more specific knowledge on the behavioral context is required to understand the role built environment plays in these processes. A better understanding of the behavioral contexts comprising LTPA can likewise help in the interpretation of related health outcomes, such as associations between overall PA and the neighborhood built environment [58] or the weak associations between body mass index, obesity, and the neighborhood availability of recreational facilities [61].

### Study strengths and limitations

The study strengths include the use of a relatively large sample of georeferenced data collected with public participation mapping. This method allowed us to locate moderate-to-vigorous LTPA separately from other PA domains. In addition, the study design followed a socio-ecological framework addressing multiple levels of influence on LTPA, including intrapersonal and environmental factors. The study limitations include the use of a cross-sectional study design that prevents us from inferring causality concerning the associations between the residential environment, intrapersonal factors, and participation in LTPA. In addition, this study did not address residential self-selection [62, 63]. This can introduce bias if individuals’ physical activity preferences have directed their residential location choice. Moderate-to-vigorous LTPA was assessed with self-reported measures, which can lead to the overestimation of actual PA levels [64, 65]. Moreover, it should be noted that the modifiable areal unit problem (MAUP) poses challenges to GIS analysis using areal data [31, 32]. In this study, Euclidean buffers were used to measure environmental characteristics around the residential locations. This approach was chosen to capture exposure to green spaces and to retain the comparability of the buffers in size. However, Euclidean buffers may in some situations overestimate the accessibility of destinations typically accessed following the street-network, such as indoor sport facilities. Access to such destinations can be better captured with street-network buffers [66]. Last, due to limitations in data collection, this study did not assess the MET-minutes in each mapped LTPA destination. Future studies using participatory mapping methods to the study of recreational behavior might benefit from estimating usual time spent at the destination.

## Conclusions

This study identified and empirically tested potential reasons leading to mixed and weak associations between objectively measured neighborhood-level built environment attributes and participation in LTPA. Our results demonstrate that context-free LTPA measures have limited applicability in guiding neighborhood-level built environment interventions. While ill-suited to predicting overall participation in moderate-to-vigorous LTPA, we found the neighborhood-based measures more suitable for capturing LTPA in public open spaces, including green spaces and built open space, such as streets and walkways. Studies on the environmental correlates and determinants of LTPA focusing on LTPA in specific behavioral contexts, such as sport facilities, parks, or other public open spaces, are in a better position to establish a well-defined connection between the built environment and health behavior outcomes than studies focusing on total LTPA.

## Supplementary information

**Additional file 1.** Scales for self-efficacy and social support for PA.

**Additional file 2.** Adjusted odds and covariates of participating in moderate-to-vigorous LTPA in indoor or outdoor sport facilities, public green space, and other public open space.

## Data Availability

The datasets generated and/or analyzed during the current study are not publicly available due to privacy issues regarding the study participants’ mapped home and activity locations but are available from the corresponding author on reasonable request.

## Abbreviations

GIS: geographic information system
GPS: Global Positioning System
LTPA: leisure-time physical activity
PA: physical activity
PPGIS: public participation GIS
